# A Lightweight Zero-Trust-Based Authentication Framework for UAV Systems

**DOI:** 10.3390/s26134161

**Published:** 2026-07-01

**Authors:** Xiang Qu, Wei Ou, Mengxue Pang, Yangbo Chen, Yishan Wu, Qiuling Yue, Wenbao Han

**Affiliations:** 1 School of Cyberspace Security (School of Cryptology), Hainan University, Haikou 570228, China; 20233001362@hainanu.edu.cn (X.Q.); 24120854120002@hainanu.edu.cn (M.P.); a1299596266@163.com (Y.C.); 20223004988@hainanu.edu.cn (Y.W.); yueqiuling@hainanu.edu.cn (Q.Y.); 2 Department of Information, Beijing City University, Beijing 100083, China; 3 Jiangsu Variable Supercomputer Technology Co., Ltd., Wuxi 214100, China; 4 Institute of Information Engineering, Chinese Academy of Sciences, Beijing 100093, China; 994338@hainanu.edu.cn

**Keywords:** UAV, zero trust, SM9, rotor acoustic fingerprint, dynamic trust evaluation

## Abstract

To address identity cloning, credential leakage, and delayed authorization in unmanned aerial vehicle (UAV) systems operating in open airspace, we propose a zero-trust authentication and authorization framework. SM9 identity-based mutual authentication establishes a traceable digital identity anchor. On-demand rotor acoustic verification then checks whether the current platform matches the registered identity. A Beta-distribution trust model converts authentication, verification, and behavioral evidence into dynamic authorization decisions. Experimental results show that the acoustic module blocks 104 of 110 spoofing samples. Under identity-cloning attacks, the attacker’s trust value decreases from about 0.97 to 0.27 and falls below the isolation threshold of 0.4. Compared with continuous acoustic authentication, on-demand triggering reduces authentication activation frequency, average inference latency, and normalized energy consumption by 86.8%, 83.6%, and 83.0%, respectively. These results indicate that the framework links identity confirmation, entity verification, and continuous authorization under resource-constrained UAV conditions.

## 1. Introduction

Unmanned aerial vehicle (UAV) systems are dynamic collaborative platforms that include UAVs, ground control terminals, edge gateways, and mission services. They support sensing, communication, inspection, delivery, and emergency response in mission airspace. As UAV services expand, the European Union has proposed a large-scale drone service market [1], the United States is promoting the safe integration of UAVs into the national airspace system [2], and China is advancing deployment of unmanned and intelligent aviation equipment [3]. These platforms increasingly operate within space–air–ground integrated networks (SAGINs) that combine satellites, high-altitude platforms, and ground infrastructure, introducing additional challenges in cross-domain coordination and dynamic resource management [4,5]. UAV systems are therefore becoming important for mission execution, but they also face open wireless links, dynamic access relations, and time-varying authorization requirements.

However, UAV security cannot rely only on flight control, communication links, or a single authentication algorithm. It requires an integrated mechanism for identity confirmation, entity verification, and authorization control. UAV platforms usually operate in open wireless environments, where node positions, link relations, and mission privileges change over time. A comprehensive UAV security survey shows that UAV systems face multiple attack surfaces, including hardware, software, communication protocols, and onboard components [6]. The system must therefore answer three questions: whether a node can access the system, whether the admitted node is still the registered entity, and whether it should retain its current privileges.

In this paper, a UAV-oriented authentication framework denotes a coordinated security architecture for mission UAVs, gateways or ground control terminals, key-management entities, and authorization services. Conventional authentication frameworks for wireless sensor networks usually organize security around node registration, mutual authentication, session-key establishment, and re-authentication for secure data sessions [7]. These functions remain necessary in UAV systems, but they are not sufficient on their own. UAVs operate as mobile aerial platforms that may move across gateways, experience intermittent wireless links, request mission-dependent privileges, and remain physically exposed to capture or cloning. A UAV authentication framework must therefore link digital credential validation with physical entity consistency and post-access authorization control.

These risks shift UAV security from one-time admission control to continuous authorization after access. Traditional perimeter-based defenses are insufficient for systems with fluid boundaries, fast link changes, and time-varying mission privileges. Zero-trust architecture fits this setting. It replaces static boundary defense with fine-grained control over users, devices, and resources, and it does not grant trust because of network location or asset ownership alone. Instead, it requires explicit authentication and authorization before access is granted [8]. In UAV scenarios, nodes may disconnect, reconnect, access the system across domains, or be physically captured. The system must therefore verify digital identity, entity consistency, and behavioral state after access. Recent UAV authentication studies have extended from single-domain admission to cross-domain access, device-side anti-cloning, and blockchain-assisted verification [9,10,11]. These studies support identity verification in open flight environments, but they mainly focus on access authentication or cross-domain verification. Post-access entity verification and its translation into continuous authorization remain insufficiently studied.

Existing studies related to this requirement can be grouped into three research directions. The first direction focuses on digital identity authentication and reduces certificate management and access overhead through identity-based cryptography or lightweight handshakes. SM9 provides a standardized foundation for identity-based cryptography [12] and has been applied to UAV access authentication [13]. The second direction focuses on entity identification. It uses physical features such as radio-frequency fingerprints or rotor acoustic fingerprints to check whether the current flight platform matches the registered identity. RF fingerprints can identify UAV telemetry radios [14]. Acoustic fingerprints can authenticate UAVs using rotor acoustic features [15] and have been extended to open-set recognition and mobile deployment [16]. The third direction focuses on continuous authorization and adjusts node privileges through trust models and access-control policies. These research directions address digital credentials, physical entities, and authorization adjustment, respectively. In UAV missions, however, the three often need to operate together.

The key difference among existing solutions is the trade-off among security, real-time performance, and resource overhead. Pure cryptographic authentication has low communication overhead and is suitable for dynamic access scenarios. However, after credential leakage or platform cloning, it cannot prove that the current flight entity is still trustworthy. Physical-layer verification improves entity-consistency assessment, but sampling conditions, background noise, and model inference introduce extra overhead [17,18]. The Beta reputation model provides a historical-behavior basis for continuous authorization [19]. Yet dynamic authorization must also suppress intermittent malicious behavior [20,21] and incorporate multi-attribute trust computation [22] and context-aware access control [23]. Thus, sustained UAV security depends not on a single authentication technology, but on a zero-trust authorization loop that coordinates multiple types of evidence according to risk.

To address these issues, this paper proposes a zero-trust authentication and authorization method for UAVs. It integrates SM9 lightweight access authentication, rotor acoustic fingerprint verification, and Bayesian dynamic trust evaluation. SM9 first establishes a digital identity anchor and reduces certificate management overhead. Rotor acoustic fingerprint verification is then triggered by high-risk authorization requests, trust fluctuations, or behavioral anomalies. It checks whether the current physical entity matches the registered identity. Finally, a Bayesian trust model combines authentication results, entity verification results, and historical behavior to decide whether to maintain authorization, downgrade authorization, or isolate the node.

The main contributions of this paper are as follows:

1. We construct a zero-trust authentication and dynamic authorization framework for UAVs. The framework links digital identity authentication, entity verification, and session-level dynamic authorization, preventing access authentication from being treated as long-term trust.
2. We design a lightweight bidirectional identity handshake protocol based on SM9 and use it as the digital identity anchor for UAVs to reduce certificate management and authentication overhead in highly dynamic access scenarios.
3. We propose a continuous authorization mechanism that combines acoustic fingerprint verification with Beta trust updating. The mechanism jointly evaluates physical consistency and long-term behavioral trustworthiness under credential leakage or identity cloning, thereby improving authorization robustness and resource efficiency.

The remainder of this paper is organized as follows. Section 2 reviews related work on UAV identity authentication, physical-layer authentication, and dynamic authorization. Section 3 presents the proposed zero-trust authentication and dynamic authorization method. Section 4 analyzes the security of the scheme from the perspectives of cryptographic authentication and dynamic authorization. Section 5 evaluates SM9 access overhead, acoustic fingerprint verification performance, and dynamic authorization effectiveness through experiments. Section 6 concludes the paper and discusses future work.

## 2. Related Work

Classical authentication frameworks in wireless sensor networks and IoT systems provide a useful baseline for defining UAV authentication. They typically involve a sensor or user node, a base station or gateway, and a trusted authority that supports initial registration, mutual authentication, key agreement, and re-authentication for later secure data sessions. For example, Bilal and Kang proposed a secure mobile sensor-network authentication protocol in which a mobile node first completes initial authentication with the base station and then uses a re-authentication ticket to establish multiple data sessions [7]. This model addresses data-session security and repeated authentication overhead, but it does not explicitly address whether an authenticated digital identity remains bound to the same mobile physical platform during a mission. UAV systems therefore require additional support for cross-domain gateway access, intermittent links, mission-dependent privileges, and entity-consistency verification after admission.

Existing research on continuous UAV security can be divided into three links: digital identity authentication in the access phase, entity verification in the session phase, and dynamic authorization for privilege adjustment. A comprehensive UAV security survey shows that UAV systems involve multiple attack surfaces, including hardware, software, and communication protocols [6].

Unauthorized access through man-in-the-middle interception and denial-of-service disruption are representative threats against UAV communication links [24,25,26].

Digital identity authentication determines whether a node has legitimate admission credentials. Entity verification checks whether the current flight platform still matches the registered identity. Dynamic authorization decides which privileges the node can obtain in later interactions.

In the broader network context, UAVs increasingly operate within space–air–ground integrated networks (SAGINs) that combine satellites, aerial platforms, and ground infrastructure. Huang et al. studied joint offloading and resource allocation for hybrid cloud and edge computing in SAGINs, where UAVs must coordinate task scheduling within the limited coverage time of LEO satellites [4]. The same authors further proposed a hierarchical federated edge learning framework in which UAVs serve as edge servers and LEO satellites provide multi-layer aggregation [5]. These results indicate that UAV security mechanisms must account for dynamic resource constraints and multi-domain coordination in addition to credential verification.

Recent studies have moved from simple access authentication to cross-domain cooperation, device-side anti-cloning, and lightweight deployment. Xie and Wang proposed a PUF-based cross-domain anonymous authentication protocol. It enables UAV-ground-station authentication without involving the control center in every run and uses PUFs to mitigate capture and forgery risks [9]. For multi-server Internet of Drones environments, Ju et al. combined blockchain, PUF, and Hash-based authentication to reduce communication overhead among users, servers, and UAVs [10]. Qiao et al. proposed BCDAIoD, which uses blockchain to support cross-domain authentication and reduce the verification burden of ground stations [11]. These studies consider efficiency, mobility, and device-side security. However, their results mainly support access authentication or session-key establishment, and are not fully extended to entity verification and continuous authorization.

At the access-authentication layer, existing studies mainly address low-overhead identity establishment, cross-domain access, and device-side credential protection. Xie and Wang used PUFs to protect UAV-side key materials against capture and forgery [9]. Ju et al. used blockchain records together with PUF and Hash operations to reduce authentication overhead in multi-server IoD scenarios [10]. Qiao et al. used blockchain to support authentication migration among ground stations [11]. These schemes make digital identity authentication more suitable for open and dynamic UAV systems, but they still focus on access qualification. SM9 is an identity-based cryptographic system and has been incorporated as the SM9 mechanism in ISO/IEC 18033-5:2015/Amd 1:2021 [12]. Su et al. applied SM9 to cellular-connected drone authentication and combined it with on-chain identity records for identity authentication, key establishment, and auditing [13].

Existing access-authentication mechanisms often assume that a node can remain trusted for a period after authentication. This assumption is weak when keys are leaked, a UAV is captured, or a same-model platform is cloned [24]. In such cases, an attacker may hold valid digital credentials without being the registered entity. Strengthening digital authentication alone cannot solve this session-stage entity-consistency problem. Independent physical-layer evidence is therefore required.

Physical-layer identity authentication bridges the gap between digital credentials and the real flight platform. RF fingerprints can use transient telemetry-radio signals and RF front-end differences to identify UAVs. Tian et al. verified the feasibility of RF footprints for UAV identification in a same-model telemetry-radio scenario [14]. Such methods do not need to modify upper-layer identity-authentication processes and can provide entity-identification evidence under external observation conditions. Their performance, however, is often affected by receiving distance, channel quality, antenna posture, background noise, and differences among devices of the same model. Vision, radar, and telemetry-consistency checking can also be used for entity discrimination, but they are respectively constrained by line of sight and illumination, dedicated hardware cost, and the possibility that telemetry data may be relayed or forged.

Acoustic fingerprints provide another physical-layer authentication method for UAVs. Diao et al. built an MFCC-based acoustic fingerprint framework and cast UAV authentication as acoustic feature matching [15]. DroneAudioID further verified acoustic fingerprint authentication in open-set recognition and mobile deployment [16]. Liu et al. studied acoustic recognition robustness in complex low-SNR environments [17]. Xu and Luo showed that RF, acoustic, radar, and vision methods all involve trade-offs among detection distance, environmental noise, model complexity, and deployment cost [18]. Zhang et al. combined federated learning with zero-trust principles and proposed LSNet, a lightweight spectrogram-based network for UAV authentication using RF signals. The reported closed-set accuracy is 85.67% with only 0.23 M parameters [27].

For continuous security control during the session phase, dynamic trust and access-control studies mainly focus on how to adjust node privileges according to historical behavior, evidence fusion, and evolving attack patterns. Related methods include extensions of static RBAC/ABAC rules, threshold counting, multi-source evidence fusion, learning-based risk scoring, and probabilistic trust models. The reputation framework proposed by Ganeriwal et al. provides a classical basis for Beta-distribution-based trust evolution [19]. Chae et al. discussed mitigation of on-off attacks from the perspective of suppressing trust recovery [20]. Mendoza and Kleinschmidt verified the effectiveness of a distributed trust mechanism in mitigating on-off attacks in IoT scenarios [21].

Kannan et al. further showed that intra-daily variability analysis can detect on-off attacks that exploit trust-recovery mechanisms by alternating between normal and malicious behavior [28].

In more recent UAV system research, Hu et al. proposed GDM-DTM, which supports malicious-node detection through multi-attribute dynamic trust computation [22]. The survey on IoT access control by Ragothaman et al. emphasizes the importance of dynamic policies, context awareness, and authorization adjustment [23]. These studies indicate that dynamic trust and context-aware authorization can support behavior-evidence accumulation and privilege adjustment during the session phase.

In summary, existing research supports digital identity authentication, physical-layer entity identification, and dynamic authorization, but the three links remain weakly coupled. Digital authentication proves credential legitimacy but not physical consistency. Physical-layer identification distinguishes entities but often does not define when it should be triggered or how it should affect authorization. Dynamic trust accumulates behavioral evidence but is rarely coupled with access authentication and entity verification. UAV zero-trust authentication therefore requires a session-level loop that integrates access authentication, entity verification, and dynamic authorization.

## 3. System Scheme

### 3.1. Overall Architecture

This paper considers zero-trust authentication and dynamic authorization for UAV missions. The system includes multiple UAVs, gateways, or ground control terminals, a trusted key generation center (KGC), and on-demand acoustic monitoring terminals. UAVs form time-varying cooperative relations during mission execution. The main challenge is that node locations, communication links, and authorization requests change with the mission. A single access authentication cannot guarantee entity consistency or stable authorization in later sessions.

The system uses three types of evidence. Digital identity evidence $A_{i}\left(t\right)$ comes from the SM9 handshake, identity whitelist, and revocation status. It indicates whether a node holds legitimate digital credentials. Entity verification evidence $R_{i}\left(t\right)$ comes from on-demand rotor acoustic sampling and classification. It indicates whether the current flight platform matches the registered identity. Authorization behavior evidence $B_{i}\left(t\right)$ comes from historical interactions, authorization requests, and abnormal events. It describes session-stage trustworthiness. Since acoustic sampling and model inference introduce energy and latency overhead, the system triggers entity verification only for high-risk authorization requests, near-threshold trust values, or abnormal behavioral changes. The trigger variable is denoted by $I_{i}^{trig}\left(t\right)$. The three types of evidence are then fed into the Bayesian trust update and dynamic authorization module.

The general symbols used in this section are defined in Table 1.

The threat model considers three categories of attackers with increasing capability [24].

**Type I: External attacker without valid credentials.**

The attacker monitors, intercepts, tampers with, or replays messages over the wireless link, but does not possess a legitimate private key [24,25]. Without the private key, the attacker cannot generate a valid SM9 signature for a fresh challenge. Forging a valid authentication message under these conditions reduces to breaking the existential unforgeability of the SM9 signature scheme, which is assumed to be computationally infeasible. This will be formally proven in Theorem 1 (Section 4.1).

**Type II: Identity-cloning attacker with stolen credentials.**

The attacker obtains a legitimate credential through physical capture, remote compromise, or key leakage, and then requests high-risk privileges with a valid digital identity. The digital identity is valid, but the physical entity is inconsistent with the registered UAV. Because the motor, propeller, and fuselage structure of a cloned platform are difficult to reproduce, the rotor acoustic fingerprint of the cloned platform usually does not match the registered template. As formalized in the later probability analysis, the system is broken only when the attacker simultaneously satisfies valid digital identity and passes acoustic verification: $P_{break}=P_{miss}+P_{hit}\cdot P_{spoof}$, where $P_{miss}$ is the false rejection rate, $P_{hit}$ is the false acceptance rate, and $P_{spoof}$ is the probability that the cloned platform produces a matching acoustic fingerprint. Since $P_{spoof}$ is small for same-model clones, acoustic verification substantially reduces $P_{break}$ compared with digital-only authentication.

**Type III: Intermittent attacker with on-off strategy.**

The attacker may hold valid credentials and pass initial authentication. It launches abnormal authorization requests, flooding, or black-hole behavior during critical periods, and then behaves normally during dormant periods to recover its trust value. As analyzed later in Theorem 5 and the trust-recovery expression, the Bayesian trust model with temporal decay and asymmetric penalty ($\omega_{pun}\gg \omega_{res}$) ensures that trust recovery is much slower than trust decline: ${Trust}_{t+k}\approx \left(\lambda^{k}\alpha +k\cdot \omega_{normal}\right)/\left(\lambda^{k}\alpha +\beta_{high}+k\cdot \omega_{normal}\right)$. The historical malicious record $\beta_{high}$ remains in the denominator, preventing the attacker from restoring high-privilege authorization through short-term normal behavior.

For flooding or concurrent-permission attempts, the framework applies conservative authorization control rather than privilege escalation. A burst of high-risk requests within a short time window is treated as abnormal behavioral evidence in the dynamic trust state. When the same digital identity appears at different gateways or at incompatible locations almost simultaneously, the event is regarded as a contextual inconsistency instead of two independent legitimate sessions. Under these conditions, the gateway can downgrade the requested privilege, trigger acoustic re-verification when entity consistency must be checked, or isolate the node after the trust value falls below the threshold. This response is defined at the authorization layer for requests that reach the gateway, whereas high-volume traffic filtering and network-layer DDoS mitigation are handled by lower-layer communication defenses.

The design objective is to form a closed loop from access to authorization. In the access phase, SM9 establishes a traceable digital identity anchor. In the session phase, acoustic fingerprint verification is triggered by risk-related events to check entity consistency. In the authorization phase, digital identity evidence, entity evidence, and historical behavior are combined to update the Bayesian trust value. The updated trust value is used to maintain, downgrade, or isolate node privileges.

**Figure 1:**
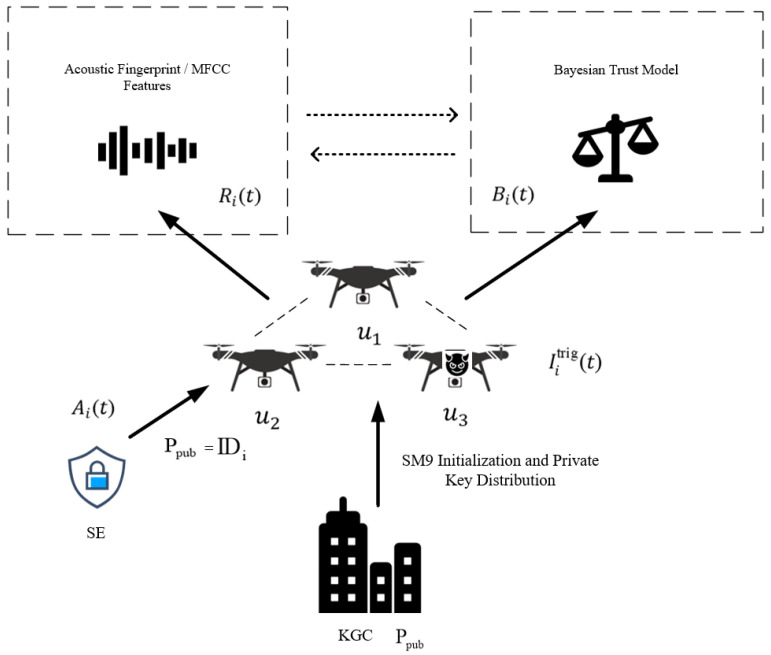
Schematic diagram of the overall system model. Arrows denote information, evidence, and authorization-control flows among modules. UAV denotes unmanned aerial vehicle, GCS denotes ground control station, KGC denotes key generation center, and SE denotes secure element.

The overall system architecture and core modules are shown in Figure 1. The overall model consists of four core dimensions: identity and cryptographic management, UAV cooperative operation, acoustic entity verification, and dynamic authorization. At the foundation of the system, the trusted key generation center (KGC) serves as the security foundation. It initializes the system based on the SM9 algorithm, assigns each UAV identity a corresponding public key, and provisions private keys to the secure elements (SEs) of the nodes. This enables lightweight SM9 identity-based authentication without certificate transmission. At the core operating layer, multiple highly dynamic UAVs form time-varying cooperative relationships in three-dimensional airspace. Some legitimate digital identities may be abused by attackers due to key leakage or device cloning, creating an authorization risk in which the digital identity is legitimate but the physical entity is not trustworthy.

To continuously confirm node identity and authorization status, the system introduces a three-layer mechanism: digital identity, entity status, and trust-based authorization. SM9 establishes digital identity in the access phase. Rotor acoustic fingerprints re-verify entity status in the session phase. The Bayesian trust model updates the final authorization state from previous authentication results and historical interactions. The system therefore answers three questions during operation: whether the node has valid digital credentials, whether the current physical entity matches those credentials, and what privileges the node should currently hold.

### 3.2. Core Modules

To address unauthorized access, identity cloning, and delayed authorization in open airspace, this paper proposes a continuous authentication and dynamic authorization framework. Consistent with zero-trust design, the framework does not treat initial admission as lasting trust. Instead, it builds a loop of identity admission, entity verification, and dynamic authorization.

**Figure 2:**
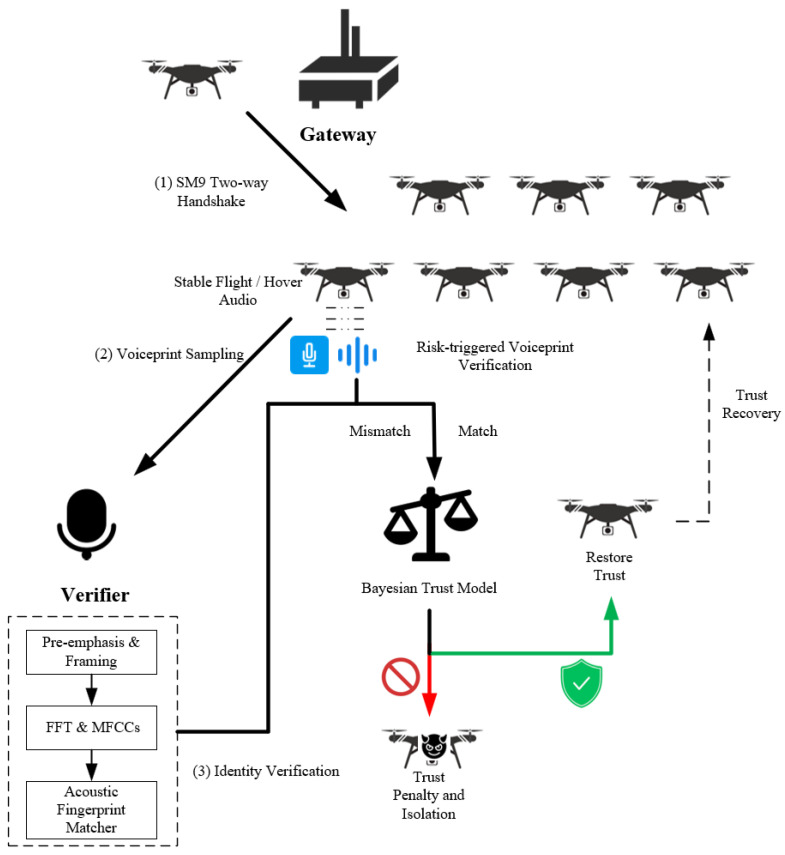
Architecture of the system defense workflow. Arrows indicate the sequential flow from access authentication to risk-triggered entity verification, trust update, and authorization response; colored blocks distinguish identity, entity, and authorization functions.

As shown in Figure 2, the framework follows the process of “SM9 mutual handshake–risk-triggered acoustic fingerprint verification–Bayesian trust update”. The gateway first completes an SM9 mutual handshake with the UAV to verify digital identity and establish a digital identity anchor for the session. When an authenticated node triggers a high-risk authorization request, trust fluctuation, or sensitive mission condition, the system starts acoustic sampling. The collected rotor audio is processed by pre-emphasis, framing, FFT, and MFCC feature extraction, and is then matched with the registered acoustic template. A successful match enters the Bayesian trust model as positive evidence. A mismatch triggers trust penalty, privilege reduction, or node isolation. Thus, digital identity authentication, entity verification, and dynamic authorization form a closed loop during the session.

A key design choice that distinguishes this framework from continuous acoustic authentication is the on-demand triggering mechanism. Continuous monitoring requires the acoustic module to sample, extract features, and classify at every time slot, which incurs high energy consumption and inference latency that are impractical for resource-constrained UAV platforms. In contrast, the proposed framework triggers acoustic verification only when the risk indicator exceeds a threshold. Specifically, the trigger variable $I_{i}^{trig}\left(t\right)$ is set to 1 when at least one of the following conditions holds: the node requests a high-risk control privilege, the trust value approaches the safety threshold $\theta_{th}$, or the behavioral pattern deviates from the historical baseline. When $I_{i}^{trig}\left(t\right)=0$, the system relies solely on digital identity and accumulated trust, incurring no additional acoustic overhead. This design converts the verification problem from a per-slot classification task into a risk-driven selective activation, reducing the average number of acoustic inferences while preserving the ability to detect identity-cloning attacks at critical moments. Formally, the trigger condition is defined as $I_{i}^{trig}\left(t\right)=I\left({\mathrm{risk}}_{i}\left(t\right)>\delta \right)$, where ${\mathrm{risk}}_{i}\left(t\right)$ is a composite risk indicator and δ is the activation threshold. The detailed composition of ${\mathrm{risk}}_{i}\left(t\right)$ is given in Section 3.4.

### 3.3. Lightweight SM9-Based Mutual Identity Handshake Protocol

The key symbols used in this section are defined in Table 2.

This section presents a lightweight SM9-based mutual identity handshake protocol. The protocol does not grant long-term trust at access time. It establishes a traceable, verifiable, and auditable digital identity anchor for each UAV node without transmitting public-key certificates. This anchor is later used for acoustic verification and Bayesian dynamic authorization. Since SM9 binds identity strings to cryptographic key material, the protocol removes certificate transmission and verification. A nonce-exchange mechanism is used to prevent replay attacks.

Access authentication is constrained by UAV operation. Nodes are numerous, links change rapidly, and disconnection and reconnection may occur during missions. Traditional ECC/RSA certificate systems are mature, but certificate transmission, chain verification, and revocation synchronization increase communication and management costs. Hash/XOR and pre-shared-key mechanisms reduce computation, but they are less suitable for revocation management, mutual signature verification, and post-event traceability at scale. PUF-based schemes strengthen hardware uniqueness, but require additional hardware and stable challenge-response modeling. SM9 derives the public key directly from the identity identifier, avoids certificate transmission during access, and supports mutual signatures and identity binding. Therefore, this paper uses SM9 as the access-stage digital identity anchor, not as sufficient evidence of continuous session trust.

**Figure 3:**
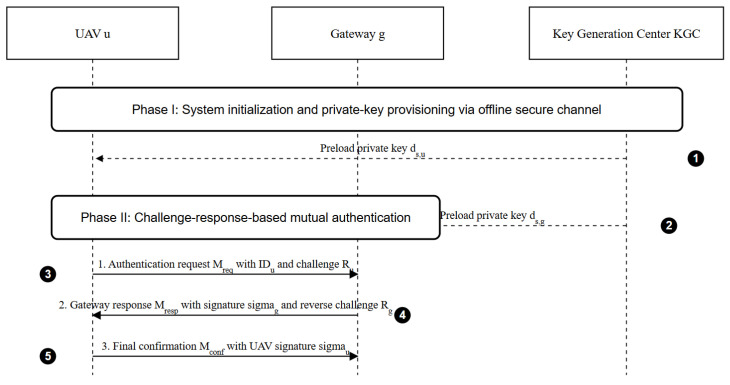
SM9-based mutual identity authentication protocol flow. Dashed arrows indicate offline private-key provisioning, and solid arrows indicate online challenge-response authentication messages.

The protocol consists of two stages: system initialization and mutual handshake interaction, as shown in Figure 3.

(1) System initialization and private-key provisioning. Before UAV deployment, the KGC configures the system parameters. A bilinear pairing $e:G_{1}\times G_{2}\to G_{T}$ is defined, the master private key s∈[1,N−1] is selected, and the master public key $P_{pub}=s\cdot P_{2}$ is published. For a legitimate UAV *u* and gateway *g* in the network, the KGC computes the corresponding private keys $d_{s,u}$ and $d_{s,g}$ according to their identity identifiers, such as $ID_{u}$ and $ID_{g}$. To ensure physical security of the keys, the private keys are provisioned into the onboard SE through an offline secure channel and cannot be read externally. This provides a strong physical binding between identity and key.
(2) Challenge-response-based mutual handshake. When UAV *u* requests network access, it must perform strict mutual identity confirmation with gateway *g*. To achieve replay resistance and forgery resistance without transmitting digital certificates, this paper designs the following three-step interaction.

Step 1: Authentication request and challenge initiation. UAV *u* first generates a high-entropy random number $R_{u}\in {\left\{0,1\right\}}^{k}$ as a challenge value, encapsulates it with its plaintext identity $ID_{u}$ into the authentication request message $M_{req}$, and sends the message to the gateway. This step transmits only the necessary identity and nonce and does not include a public-key certificate, significantly reducing communication payload.

(1) $$M_{req}=\left\{ID_{u},R_{u}\right\}$$

Step 2: Gateway response and reverse challenge. After receiving the request, gateway *g* first checks the local whitelist to confirm the legitimacy of $ID_{u}$. If the check passes, the gateway generates its own random number $R_{g}$ as a reverse challenge and uses its private key $d_{s,g}$ to sign the concatenated message containing both nonces and identities, producing $\sigma_{g}=Sign(d_{s,g},R_{u}\;\|\;R_{g}\;\|\;ID_{u})$. The gateway then returns the response message $M_{resp}$ to the UAV. Because the signature includes $R_{u}$, this step proves gateway freshness and effectively prevents replay attacks.

(2) $$M_{resp}=\left\{ID_{g},R_{g},\sigma_{g}\right\}$$

Step 3: UAV signature verification and final confirmation. After receiving the response, UAV *u* uses the system master public key $P_{pub}$ and the gateway identity $ID_{g}$ as the public key and calls $Verify(P_{pub},ID_{g},\sigma_{g})$ to verify the signature. If verification passes, the UAV confirms that the gateway identity is legitimate and that the message has not been tampered with. It then uses its own private key $d_{s,u}$ to sign the gateway challenge $R_{g}$, generates the confirmation signature $\sigma_{u}=Sign\left(d_{s,u},R_{g}\;\|\;ID_{g}\right)$, and sends it back to the gateway.

(3) $$M_{conf}=\left\{ID_{u},R_{g},\sigma_{u}\right\}$$

After receiving $M_{conf}$, the gateway verifies the validity of $\sigma_{u}$. If verification succeeds, mutual identity authentication is completed. The protocol not only enables millisecond-level fast access, but also provides identity traceability for subsequent acoustic verification and dynamic authorization. Any later authorization request or interaction record judged as abnormal can be reliably traced to a specific UAV ID through its attached signature, supporting precise trust penalties for malicious nodes in the Bayesian model.

From the perspective of the security function, the protocol has three effects. First, using the identity identifier directly as the public key avoids the additional communication overhead caused by certificate transmission, certificate-chain verification, and revocation-list synchronization. Second, mutual signatures are bound to random challenges, making it difficult for attackers to complete replay attacks by intercepting historical authentication messages. Third, all key access requests can be traced to a specific digital identity through signature records, providing a basis for cloned-identity detection, privilege reduction, and traceability.

### 3.4. Acoustic Fingerprint-Based Physical-Entity Verification

The key symbols used in this section are defined in Table 3.

Digital identity authentication confirms whether a node holds legitimate credentials. It does not prove that the current flight entity is trustworthy after physical replacement, same-model impersonation, or credential leakage. Existing studies show that UAV rotor sound contains individual differences caused by manufacturing tolerances in motor windings, propeller blade pitch, and fuselage resonance structure. Even among UAVs of the same model and batch, slight variations in these components produce distinguishable spectral patterns in the emitted sound. Such differences can serve as acoustic fingerprints for distinguishing UAVs of the same model [15,29]. Based on this feature, this paper introduces rotor acoustic verification in the session phase. UAV flight sound is used as entity evidence beyond digital credentials. It checks whether the requesting platform is still the UAV registered to that identity and supports subsequent dynamic authorization.

SM9 confirms legitimate identity credentials, but credential legitimacy does not prove that the current flight platform is still the registered entity. Identity-cloning attacks exploit this gap. An attacker may reuse or relay a digital identity, but it is difficult to replicate the rotor acoustic response formed by the motor, propeller, and fuselage structure. Therefore, this paper uses rotor acoustic fingerprints as entity verification evidence in the session phase. Compared with RF fingerprints, acoustic observations are less dependent on wireless channels and antenna posture. Compared with vision- and radar-based schemes, they require less dedicated hardware and fewer line-of-sight constraints. Compared with telemetry-consistency checking, they provide entity-side evidence outside digital data. Acoustic verification is still affected by background noise, sampling window, and inference overhead. Thus, it is not used to replace access authentication or as an always-on detector. It provides additional physical confirmation when high-risk authorization requests, trust fluctuations, or behavioral anomalies occur.

In the registration phase, the system establishes an acoustic reference template $V_{U}$ for each legitimate UAV identity $ID_{U}$. The template is generated from multiple rotor-audio segments collected while the UAV is hovering stably or flying at low speed, and is stored together with its digital identity, airframe number, and propeller status. The audio-processing procedure includes preprocessing, effective frequency-band preservation, framing and windowing, MFCC feature extraction, and template generation. Considering that rotor acoustic energy is mainly concentrated in the low- and mid-frequency range, the system preferentially retains the 0–8 kHz band. The audio is then divided into overlapping frames of 25 ms with a 10 ms hop size, and each frame is multiplied by a Hamming window to reduce spectral leakage. The MFCC extraction pipeline consists of the following steps: (1) compute the discrete Fourier transform of each windowed frame to obtain the power spectrum; (2) apply a Mel-scaled filterbank to the power spectrum, where the Mel scale maps physical frequency to perceived pitch with finer resolution at low frequencies; (3) take the logarithm of each filterbank output to compress the dynamic range; and (4) apply the discrete cosine transform (DCT) to the log-filterbank energies, retaining the first 13 coefficients as the feature vector for each frame. The frame-level vectors are then summarized into a segment-level descriptor by computing the mean and standard deviation across all frames, forming a 26-dimensional acoustic fingerprint vector. MFCC can describe the spectral envelope of rotor sound with low computational cost, making it suitable as a lightweight entity verification module deployed at the gateway side or a ground monitoring terminal. In addition to MFCC, alternative features such as power spectral density (PSD), Gammatone frequency cepstral coefficients (GFCC), and linear predictive coding (LPC) coefficients have been studied for acoustic UAV recognition. Mehfuz et al. compared LPC, LPCC, PSD, MFCC, and GFCC for authenticating DJI Phantom 4 Pro and Mavic Pro platforms using SVM classifiers (DJI, Shenzhen, China), and reported that PSD achieved 100% accuracy while MFCC reached 94.28% [29]. This paper adopts MFCC because it provides a favorable balance between discriminability and computational efficiency for the rotor-sound verification scenario.

During operation, acoustic verification is triggered on demand rather than through continuous monitoring. The trigger variable is denoted by $I_{i}^{trig}\left(t\right)$. When a node performs ordinary telemetry, status reporting, or low-risk communication, the system mainly maintains authorization according to digital identity authentication results and historical trust status. When a node requests high-risk control privileges, enters a sensitive mission area, has a trust value close to the safety threshold, or shows abnormal behavioral changes, the system sets $I_{i}^{trig}\left(t\right)=1$. Formally, the trigger condition can be expressed as:

(4) $$I_{i}^{trig}\left(t\right)=I\;\left({\mathrm{risk}}_{i}\left(t\right)>\delta \right)$$

where ${\mathrm{risk}}_{i}\left(t\right)$ is a composite risk indicator derived from the authorization-request level, the distance between ${Trust}_{i}\left(t\right)$ and the isolation threshold $\theta_{th}$, and the deviation of the current behavioral pattern from the historical baseline, and δ is the activation threshold.

The gateway-side or ground monitoring terminal then collects a short-window rotor audio segment and starts entity verification. This mechanism restricts the use of the acoustic module to key authorization scenarios and avoids the energy, latency, and computation overhead caused by continuous sampling and continuous inference.

The acoustic verification module consists of four functional units. First, the audio acquisition unit obtains a short rotor-sound segment of the current flight platform from the monitoring terminal and performs denoising, normalization, and effective band extraction. Second, the feature extraction unit performs framing, windowing, and MFCC computation on the audio segment to form the current acoustic feature vector. Third, the identity matching unit compares the current acoustic vector with the registered template $V_{U}$ and determines whether it is consistent with the node’s claimed digital identity $ID_{U}$. Specifically, the matching unit computes the cosine similarity between the current feature vector $x_{i}^{phy}\left(t\right)$ and the registered template $V_{U}$, defined as sim(x,V)=x·V/(∥x∥·∥V∥). If the similarity score exceeds a preset matching threshold τ, the current entity is considered consistent with the registered identity. This threshold-based comparison is computationally lightweight and does not require a trained classifier at the gateway side, which supports the on-demand triggering design. For scenarios involving more than two UAV models or requiring open-set rejection, a multi-class SVM, or an out-of-distribution detection layer can replace the cosine-similarity comparator without changing the overall verification framework. Finally, the result output unit generates the entity verification result $R_{i}\left(t\right)$ and sends it to the subsequent trust-update and authorization-decision process.

If the current acoustic fingerprint matches the registered template corresponding to the claimed identity, the system considers the digital identity and the physical entity to be consistent at the current time, sets $R_{i}\left(t\right)=1$, and injects positive evidence into the Bayesian trust model. If the current acoustic fingerprint does not match the registered template, or is classified as an unknown entity, spoofing entity, or background anomaly, the system sets $R_{i}\left(t\right)=0$ and injects negative evidence into the trust model. At this point, the system does not rely on a single acoustic result to make a permanent decision. Instead, it combines historical behavior, authorization-request type, and the current trust value to maintain authorization, downgrade authorization, or isolate the node, thereby reducing misjudgment risks caused by noise, distance variation, or short-term sampling-quality degradation.

In the proposed scheme, acoustic verification is not a standalone authentication stage. It is a physical evidence source in the zero-trust authorization chain. Digital identity authentication checks whether a node holds legitimate credentials. Acoustic verification checks whether the current flight platform matches the registered entity. The Bayesian dynamic trust model converts digital authentication, acoustic verification, and historical behavior into authorization decisions. This design exposes the separation between digital identity and physical entity under credential leakage or identity cloning, while improving continuous trust control with limited runtime overhead.

### 3.5. Bayesian Dynamic Trust Evaluation and Dynamic Authorization

The key symbols used in this section are defined in Table 4.

After entity verification, the system does not directly equate a single verification result with the final authorization decision. Instead, it maps the digital access result, acoustic verification result, and historical interaction behavior into a unified dynamic trust state. The core purpose is to avoid two extremes. One is maintaining high privileges for a long period based only on one successful access authentication, which would allow an identity-cloning node to keep obtaining privileges in later stages. The other is permanently isolating a node immediately based on one abnormal verification result, which may over-penalize legitimate nodes in complex environments. Therefore, this paper introduces a Bayesian dynamic trust evaluation mechanism during the continuous session phase to balance rapid risk handling with the suppression of false penalties against legitimate nodes.

The dynamic authorization model must satisfy three requirements: it should absorb new authentication evidence, retain historical failure records, and run stably on resource-constrained nodes. Static RBAC/ABAC is more suitable for describing authorization boundaries and does not inherently capture risk evolution during a session. Simple threshold counting is straightforward to implement, but it is difficult to distinguish occasional failures from persistent malicious behavior and lacks a clear probabilistic meaning. D-S evidence theory has advantages in multi-source uncertainty fusion, but once it is used for continuous temporal updates, parameter setting and result interpretation become computationally and interpretively burdensome. Black-box learning models require sufficient labeled attack samples, and the basis for privilege reduction or recovery is not transparent enough. A Beta-distribution trust model maintains only positive and negative evidence parameters, has low computation and storage overhead, and can express the influence of different event types through temporal decay and asymmetric penalty. In this way, legitimate nodes still have room for trust recovery after an unnecessary verification trigger, while intermittent attacking nodes cannot quickly return to a high-privilege state through short-term normal behavior.

Specifically, the system maintains a dynamic trust state $T_{i}\left(t\right)$ for each node and uses the SM9 access result $A_{i}$, the acoustic verification result $R_{i}\left(t\right)$, and the current mission context as trust-update inputs. When a node performs only ordinary telemetry or low-risk operations, the system mainly relies on the existing trust state to maintain authorization. When the node requests high-risk control commands, enters a sensitive area, frequently switches behavior patterns, or has a trust value approaching the safety threshold, the system triggers acoustic verification and re-evaluates its authorization level. Thus, the acoustic module in this section is no longer an independent discriminator, but a key evidence source that drives trust-state transitions.

To ensure that authorization decisions have interpretable historical memory, this paper adopts a Bayesian trust model based on the Beta distribution. Let the benign evidence and malicious evidence of node $u_{i}$ at time *t* be $\alpha_{i}\left(t\right)$ and $\beta_{i}\left(t\right)$, respectively. The trust value $T_{i}\left(t\right)$ is then determined by the relative strength of the two types of evidence. If the node completes legitimate access and its recent entity verification is consistent, the system increases positive evidence. If the node shows acoustic inconsistency, authentication failure, a surge of abnormal requests, or deteriorating historical behavior, the system rapidly increases malicious evidence. At the same time, to prevent intermittent attackers from masking long-term malicious records through short periods of normal behavior, the parameter update introduces temporal decay and asymmetric penalties so that trust recovery is slower than trust decline.

At the authorization execution layer, this paper denotes the dynamic authorization decision as $D_{i}\left(t\right)$. When ${Trust}_{i}\left(t\right)$ remains within the safe interval, the system maintains or restores node privileges. When ${Trust}_{i}\left(t\right)$ drops to the warning interval, the system downgrades authorization and increases the trigger frequency of subsequent acoustic verification. When ${Trust}_{i}\left(t\right)$ further falls below the isolation threshold, the system rejects key mission requests and transfers the node into the isolation state. In other words, the core of this section is not to judge again whether the node exists, but to determine, based on evidence accumulated across time, which privileges the node can still obtain at the current moment and whether it should remain restricted.

Based on this design, the dynamic authorization problem is further formalized as a joint optimization problem of authentication reliability and authorization effectiveness under resource constraints. The goal is to maintain service continuity for legitimate nodes while causing identity-cloning nodes and intermittent spoofing nodes to cross the authorization boundary as quickly as possible, under constraints on verification frequency, inference latency, and energy overhead. The following formulas define the trust value, parameter update rule, and authorization objective function.

For the trust-update model, the trust value must be updated through Bayesian inference to address intermittent attacks. Suppose node behavior follows a Beta distribution, where $\alpha_{i}\left(t\right)$ represents accumulated benign evidence and $\beta_{i}\left(t\right)$ represents accumulated malicious evidence. The trust value is defined as the expectation of this distribution:

(5) $${Trust}_{i,t}=E\left[Beta\left(\alpha_{i,t},\beta_{i,t}\right)\right]=\frac{\alpha_{i,t}}{\alpha_{i,t}+\beta_{i,t}}$$

The parameter update follows a dynamic process with a temporal decay factor λ∈(0,1]:

(6) $$\begin{array}{cc} \alpha_{i,t} & =\lambda \cdot \alpha_{i,t-1}+\Delta \alpha_{t}\\ \beta_{i,t} & =\lambda \cdot \beta_{i,t-1}+\Delta \beta_{t} \end{array}$$

This update extends the standard Beta reputation system [19] by introducing temporal decay and asymmetric event-to-evidence conversion. In the original Beta reputation system [19], each positive interaction increments α by one unit and each negative interaction increments β by one unit, with no distinction in weight. To suppress intermittent attackers that alternate between malicious and normal behavior, this paper assigns asymmetric weights to positive and negative events. The evidence increments are computed as:

(7) $$\begin{array}{cc} \Delta \alpha_{t} & =\omega_{res}\cdot I\left(A_{i,t}=1\right)\cdot I\left(R_{i,t}=1\right)\\ \Delta \beta_{t} & =\omega_{pun}\cdot \left[I\left(A_{i,t}=0\right)+I\left(R_{i,t}=0\right)\right] \end{array}$$

where I(·) is the indicator function, $A_{i,t}$ denotes the SM9 authentication result (1 for success, 0 for failure), $R_{i,t}$ denotes the acoustic verification result (1 for match, 0 for mismatch), $\omega_{res}$ is the reward weight for positive evidence, and $\omega_{pun}$ is the penalty weight for negative evidence, with $\omega_{pun}\gg \omega_{res}$. This asymmetry ensures that trust declines rapidly when a node exhibits authentication failure or acoustic inconsistency, while trust recovery during normal behavior is deliberately slow. Combined with the temporal decay factor λ in Equation (5), historical malicious records are retained in the denominator of the trust value, preventing intermittent attackers from restoring high-privilege authorization through short-term compliance.

To more clearly describe the above evidence-fusion and authorization-decision process, Algorithm 1 presents the risk-triggered entity verification and dynamic authorization procedure.

In summary, the optimization objective of this paper is to maximize authentication reliability and authorization-decision effectiveness while satisfying the system energy budget and mission-authorization constraints:

(8) $$\begin{array}{cc} \max\; & \sum_{t=1}^{T_{\max}}\sum_{i=1}^{N}I\left(D_{i,t}=y_{i,t}\right)\\ s.t.\; & \sum_{t=1}^{T_{\max}}\sum_{i=1}^{N}C\left(u_{i},t\right)\leq B_{total} \end{array}$$

where $D_{i}\left(t\right)$ denotes the authorization decision of node $u_{i}$ at time *t*, ${Trust}_{i}\left(t\right)$ denotes its dynamic trust value, and $C_{i}\left(t\right)$ denotes the authentication and verification cost. This formal objective emphasizes continuous authentication, dynamic authorization, and rapid isolation under resource-constrained conditions.

Because the objective function involves sequential decisions over a discrete time horizon with a cumulative cost constraint, an exact solution requires enumerating all verification subsets across time steps, which is computationally intractable for real-time UAV systems. This paper therefore adopts an online risk-triggered heuristic strategy to approximate the optimal solution. At each time slot *t*, the system does not solve the full optimization over the remaining horizon. Instead, it makes a local decision based on the current trust value and the remaining budget. Specifically, when a node requests a high-risk authorization, its trust value approaches the safety threshold, or its behavioral pattern changes abruptly, the system prioritizes this node for acoustic verification. Nodes with trust values well above the threshold and stable behavioral history are not verified in the current slot, thereby conserving the budget for future time steps. This greedy prioritization reduces the per-slot decision to a threshold comparison and a budget check, both of which run in O(1) time per node. The complete procedure is summarized in Algorithm 1. Although the heuristic does not guarantee global optimality, the experimental results in Section 5 show that it achieves near-optimal isolation of identity-cloning nodes while maintaining low unnecessary verification overhead for legitimate nodes under the given energy budget.

**Algorithm 1** Risk-triggered physical re-authentication and dynamic trust authorization algorithm.**Input:** Node set U; SM9 authentication result $A_{i}\left(t\right)$; behavior evidence $B_{i}\left(t\right)$; historical trust parameters $\left(\alpha_{i}\left(t-1\right),\beta_{i}\left(t-1\right)\right)$; acoustic template $V_{U}$; decay factor λ; trust threshold $\theta_{th}$; reward weight $\omega_{res}$; penalty weight $\omega_{pun}$. **Output:** Updated trust value ${\mathrm{Trust}}_{i}\left(t\right)$; final authorization decision $D_{i}\left(t\right)$.
  1**for each** node $u_{i}\in U$ **do**  2    Initialize evidence increments: $\Delta \alpha_{i}\left(t\right)\leftarrow 0$, $\Delta \beta_{i}\left(t\right)\leftarrow 0$  3    **if** $A_{i}\left(t\right)=0$ **then**  4       $\Delta \beta_{i}\left(t\right)\leftarrow \Delta \beta_{i}\left(t\right)+\omega_{pun}$  5       $D_{i}\left(t\right)\leftarrow \mathrm{Isolate}$  6    **else**  7       Determine trigger variable $I_{i}^{trig}\left(t\right)$  8       **if** $I_{i}^{trig}\left(t\right)=1$ **then**  9          Capture acoustic signal $S_{i}^{raw}\left(t\right)$10          Extract features $x_{i}^{phy}\left(t\right)\leftarrow \mathrm{MFCC}\left(S_{i}^{raw}\left(t\right)\right)$11          Match $x_{i}^{phy}\left(t\right)$ with registered template $V_{U}$12          Obtain physical verification result $R_{i}\left(t\right)$13          **if** $R_{i}\left(t\right)=1$ **then**14              $\Delta \alpha_{i}\left(t\right)\leftarrow \Delta \alpha_{i}\left(t\right)+\omega_{res}$15          **else**16              $\Delta \beta_{i}\left(t\right)\leftarrow \Delta \beta_{i}\left(t\right)+\omega_{pun}$17          **end if**18       **else**19          **if** $B_{i}\left(t\right)$ indicates normal behavior **then**20              $\Delta \alpha_{i}\left(t\right)\leftarrow \Delta \alpha_{i}\left(t\right)+1$21          **else**22              $\Delta \beta_{i}\left(t\right)\leftarrow \Delta \beta_{i}\left(t\right)+\omega_{pun}$23          **end if**24       **end if**25       $\alpha_{i}\left(t\right)\leftarrow \lambda \alpha_{i}\left(t-1\right)+\Delta \alpha_{i}\left(t\right)$26       $\beta_{i}\left(t\right)\leftarrow \lambda \beta_{i}\left(t-1\right)+\Delta \beta_{i}\left(t\right)$27       ${\mathrm{Trust}}_{i}\left(t\right)\leftarrow \alpha_{i}\left(t\right)/\left(\alpha_{i}\left(t\right)+\beta_{i}\left(t\right)\right)$28       **if** ${\mathrm{Trust}}_{i}\left(t\right)<\theta_{th}$ **then**29          $D_{i}\left(t\right)\leftarrow \mathrm{Isolate}$30       **else if** $I_{i}^{trig}\left(t\right)=1$ and $R_{i}\left(t\right)=0$ **then**31          $D_{i}\left(t\right)\leftarrow \mathrm{Downgrade}$32       **else**33          $D_{i}\left(t\right)\leftarrow \mathrm{Maintain}$34       **end if**35   **end if**36
**end for**
37**return** ${\left\{D_{i}\left(t\right),{\mathrm{Trust}}_{i}\left(t\right)\right\}}_{u_{i}\in U}$


## 4. Security Analysis

This section formally analyzes the proposed zero-trust authentication framework from two dimensions: the robustness of the cryptographic protocol and the effectiveness of the dynamic authorization mechanism. The attacker is assumed to be capable of intercepting, tampering with, and replaying network communications, and may have obtained the long-term private keys of some legitimate nodes.

### 4.1. Security Proof of the Identity Authentication Protocol

This section analyzes the SM9-based access authentication protocol from four aspects: correctness, unforgeability, replay resistance, and traceability. The object of analysis is the three-step mutual handshake in SM9 access. The attacker is assumed to be able to monitor, intercept, tamper with, and replay authentication messages over the wireless link, and may attempt to impersonate a UAV or gateway. However, without obtaining the private key of the target entity, the attacker cannot forge a valid SM9 signature in polynomial time.

**Definition** **1** (correctness of access authentication). *If UAV U and gateway G both hold legitimate private keys generated by the KGC based on their identity identifiers, and both parties use the same public system parameters, then after executing the handshake, each party can verify the other party’s signature and accept the current access authentication.*

**Proof.**  In $M_{resp}$, the gateway uses private key $d_{s,g}$ to generate signature $\sigma_{g}$ over the session message containing $ID_{u}$, $R_{u}$, $R_{g}$, and $ID_{g}$. When the UAV verifies the signature, it uses the system master public key and gateway identity identifier $ID_{g}$ as verification inputs. Since the signature generation and verification algorithms satisfy SM9 signature correctness and $R_{u}$ in $M_{resp}$ is consistent with the nonce generated by the UAV in $M_{req}$, a legitimate gateway response can pass UAV verification. Similarly, in $M_{conf}$, the UAV uses its private key $d_{s,u}$ to generate confirmation signature $\sigma_{u}$ over the gateway challenge $R_{g}$, and the gateway completes verification using the UAV identity identifier $ID_{u}$. If both identities and random challenges are consistent, the gateway accepts the node. Therefore, under honest execution, the protocol can correctly complete mutual identity confirmation. □

**Theorem** **1.** *If the SM9 signature scheme satisfies existential unforgeability, an external attacker without a legitimate private key cannot forge a UAV or gateway identity and pass access authentication.*

**Proof.**  Suppose there exists an attacker A who can generate an $M_{conf}$ accepted by the gateway without knowing the target UAV private key $d_{s,u}$. Because the message verified by the gateway contains $ID_{u}$ and the freshly generated challenge $R_{g}$ in the current session, the attacker must provide a valid SM9 signature $\sigma_{u}$ for this fresh challenge to pass verification. If A can complete this forgery with non-negligible probability, then an algorithm can be constructed to use A to forge an SM9 signature under the target identity, contradicting the existential unforgeability assumption of SM9 signatures. Therefore, an external attacker without a legitimate private key cannot pass protocol authentication. Attacks that forge the gateway identity can be analyzed symmetrically. □

**Theorem** **2.** *The protocol can resist replay attacks against authentication messages.*

**Proof.**  $M_{req}$, $M_{resp}$, and $M_{conf}$ in the protocol are all bound to random challenges in the current session. If an attacker replays a historical $M_{resp}$, the $R_{u}$ contained in it will not match the new nonce generated by the UAV in the current session, so the UAV will reject the response. If an attacker replays a historical $M_{conf}$, the $R_{g}$ contained in it will not match the new nonce generated by the gateway in the current session, causing the freshness check before gateway verification to fail. Even if the replayed message contains a historically valid signature, the signature is bound to historical session parameters and cannot satisfy the random challenge of the current session. Therefore, replay attacks cannot grant the attacker valid access. □

**Theorem** **3.** *The protocol provides digital identity traceability for access requests.*

**Proof.**  SM9 binds the identity identifier to the public-key verification process. The gateway accepts $M_{conf}$ only if $\sigma_{u}$ can be verified under the public-key semantics corresponding to $ID_{u}$. Therefore, any request that passes access authentication can be associated with a specific $ID_{u}$ and its signature record. If subsequent acoustic verification shows that the current physical entity is inconsistent with the registered acoustic fingerprint of $ID_{u}$, the system can record the event as an anomaly of “valid digital identity but inconsistent physical entity” and perform authorization downgrading, isolation, and auditing accordingly. It should be noted that traceability is not equivalent to entity authenticity. If a legitimate private key is stolen, the attacker may still pass the digital handshake. Therefore, the experimental section further analyzes how acoustic verification and the Bayesian trust model suppress cloned identities. □

In summary, the SM9 access protocol can complete correct mutual digital identity authentication without certificates and can resist external forgery and replay attacks. Its security boundary, however, is mainly at the digital credential layer. For internal impersonation or identity cloning after credential leakage, relying only on cryptographic protocol proof is insufficient to ensure continuous security. Session-stage entity verification and dynamic authorization mechanisms must be combined.

### 4.2. Effectiveness Analysis of Zero-Trust Dynamic Authorization

This section analyzes the joint operation of SM9 access, acoustic verification, and Bayesian dynamic authorization when the attacker possesses legitimate digital credentials.

**Theorem** **4.** *The proposed scheme provides probabilistic resistance to identity-cloning attacks.*

**Proof.**  Suppose the attacker completely steals the digital identity of a legitimate node and launches a cloning attack. In this case, the attacker can pass SM9 digital identity verification, but still must pass acoustic verification to obtain mission-critical privileges. Since acoustic features originate from physical differences in the motor, propeller, and fuselage structure, even if the attacker uses a UAV of the same model, its acoustic fingerprint remains difficult to make completely consistent with that of the cloned node. □

(9) $$P_{break}=P_{miss}+P_{hit}\cdot P_{spoof}$$

Therefore, the condition for breaking the system is no longer merely obtaining legitimate credentials, but simultaneously satisfying valid digital identity, successful acoustic verification, and a dynamic trust value that has not been suppressed. In the experiment, acoustic authentication successfully blocks 104 of 110 spoofing samples, indicating that entity verification can significantly reduce the probability that an identity-cloning node obtains continuous authorization.

**Theorem** **5.** *The Bayesian trust model has convergent suppression capability against on-off attacks.*

**Proof.**  Suppose a spoofing node adopts an on-off strategy: it passes low-risk interactions normally during some periods and requests high-risk authorization during critical periods. Let the penalty weight of the Bayesian trust model be $\omega_{pun}$ and the reward weight be $\omega_{res}$, with $\omega_{pun}\gg \omega_{res}$. When acoustic verification fails, or authorization behavior is inconsistent, malicious evidence increases and the trust value drops rapidly. During the dormant phase, even if the node behaves normally, the growth rate of benign evidence is constrained by temporal decay and historical failure records. Therefore, the node cannot quickly recover high-privilege authorization through short-term normal behavior. □

(10) $${Trust}_{t+k}\approx \frac{\lambda^{k}\alpha +k\cdot \omega_{normal}}{\lambda^{k}\alpha +\beta_{high}+k\cdot \omega_{normal}}$$

Because $\beta_{high}$, the historical malicious record, remains in the denominator and $\omega_{normal}$ is relatively small, the slope of trust recovery is far smaller than the slope of trust decline; that is, trust is difficult to build but easy to destroy. Therefore, for periodic attackers, the long-term average trust value satisfies $Trust<\theta_{th}$, and the system continuously isolates them. They cannot restore high privileges simply by remaining dormant for a short period.

## 5. Experiments and Results Analysis

### 5.1. Experimental Setup

#### 5.1.1. Experimental Environment and Parameter Settings

The experiments were conducted on a server equipped with an NVIDIA GeForce RTX 3090 GPU with 24 GB of memory and an Intel Core i9 processor. The software environment was based on Python 3.8. The SM9 identity-based cryptographic algorithm was used to implement the signing and verification process in the access phase. Librosa was used to extract MFCC features from UAV rotor audio, and an acoustic classifier was used to complete entity verification. In the authorization simulation, the initial parameters of the Bayesian trust model were set to α=1 and β=1, with an initial trust value of 0.5. The trust isolation threshold was set to 0.4. The access-authentication component used protocol-level simulation statistics, while the acoustic verification and dynamic authorization components used comparative simulations under unified scenario parameters to evaluate the system’s overall performance in computation overhead, entity discrimination, and continuous authorization convergence.

The experimental environment and authentication-authorization scenario parameters are summarized in Table 5.

#### 5.1.2. Dataset and Authentication Scenario Construction

At the digital identity layer, SM9 authentication is used to establish a basis for legitimate access. At the entity layer, the Mendeley Drone Audio Dataset is used as the input to the acoustic verification module, and rotor acoustic features of different aircraft models are extracted. At the authorization evaluation layer, four types of objects are constructed: legitimate nodes, legitimate nodes subjected to additional verification, identity-cloning nodes, and intermittent spoofing nodes. These objects are used to simulate the evolution of dynamic authorization in a zero-trust environment.

The experiments consider two representative scenarios. In the legitimate scenario, a node can pass SM9 access, and its rotor acoustic fingerprint is consistent with the registered identity. In the identity-cloning scenario, an attacker steals the digital identity of a legitimate node and can apparently pass digital identity verification, but its physical entity comes from an unregistered aircraft model. This creates a conflict in which the digital identity is legitimate, but the physical entity is inconsistent. The authorization system continuously updates the node trust value accordingly and determines whether to continue granting mission-critical privileges.

#### 5.1.3. Evaluation Metrics and Baselines

The main evaluation metrics used in this paper include acoustic verification interception rate, misidentification rate, node trust-value trajectory, speed of crossing the isolation threshold, authorization recovery speed, authentication activation frequency, average inference latency, normalized energy consumption, and GPU memory usage. The comparison baselines include a one-time static authentication scheme relying only on digital identity, a continuous acoustic authentication scheme, a traditional weighted trust model, and the proposed SM9 access + acoustic verification + Bayesian dynamic authorization scheme.

### 5.2. SM9 Lightweight Access Authentication Overhead Analysis

To verify the deployability of the proposed access-authentication module in UAV systems, we conducted a protocol-level simulation analysis of the SM9-based UAV-gateway mutual identity handshake and quantitatively evaluated it from three dimensions: signing/verification latency, complete handshake latency, and message communication overhead. Unlike traditional certificate-based authentication, the SM9 identity-based cryptographic mechanism directly uses the identity identifier as the public key. No certificate chain needs to be transmitted or verified during authentication. Its advantages therefore lie mainly in shorter message length, simpler identity management, and an access path more suitable for highly dynamic node environments.

The evaluated access-authentication process contains three messages. First, the UAV sends an authentication request containing the UAV identity identifier and a random challenge value to the gateway. Then, after completing identity whitelist checking, the gateway returns its own identity identifier, a reverse random challenge, and an SM9 signature over the identities and challenge values of both parties. Finally, the UAV verifies the gateway response and generates a confirmation signature, which is returned to the gateway. This process ensures message freshness through mutual random challenges and realizes mutual identity binding through SM9 signatures.

For the SM9 access component, protocol-level simulation statistics were collected from 1000 independent access requests. The latency distributions of UAV-side signing, gateway-side verification, gateway-side signing, UAV-side verification, and the complete three-step handshake were recorded, and the mean and P95 values are reported. These results characterize the operating cost of the authentication process under a unified parameter configuration and reflect the computation and communication load of the access phase.

The SM9 access-module simulation overhead is reported in Table 6.

The results show that the average latency of the complete three-step handshake is 20.4 ms, and the estimated P95 latency is 24.3 ms. Each individual signing and verification process is stably controlled within 15 ms. For access scenarios in UAV systems, this overhead is significantly lower than that of highly complex key-agreement processes, while avoiding the certificate transmission and chain-verification cost of traditional PKI schemes. It can therefore satisfy the fast access requirements at the beginning of a mission.

Table 7 reports the communication payload of access-authentication messages. The total payload of the three-step handshake is 152 bytes, with the main overhead of Mresp and Mconf coming from the SM9 signature fields. Reporting both latency and communication overhead enables comparison with existing UAV authentication schemes under common evaluation dimensions. The current simulation results show that, while ensuring bidirectional identity binding and auditability, the module keeps the communication payload at a low level and therefore provides a stable digital identity foundation for subsequent acoustic verification and dynamic authorization.

SM9 access proves only that a node holds valid digital identity credentials; it cannot independently prove that the current flight platform is physically consistent with the registered UAV. Therefore, this paper treats SM9 as the lightweight digital identity anchor in the zero-trust framework, rather than as a complete continuous identity assurance mechanism. Rotor acoustic verification and Bayesian dynamic trust evaluation during the session phase are further used to address identity cloning and authorization-delay risks after credential leakage.

### 5.3. Acoustic Verification Performance Analysis

The acoustic verification module verifies the flight entity after a node passes SM9 access. It aims to address the problem that digital identity can be copied while the physical entity is difficult to forge synchronously. This section evaluates the acoustic module from two dimensions, verification accuracy and environmental robustness, to examine its usability in open UAV environments.

#### 5.3.1. Acoustic Fingerprint Verification Accuracy

**Figure 4:**
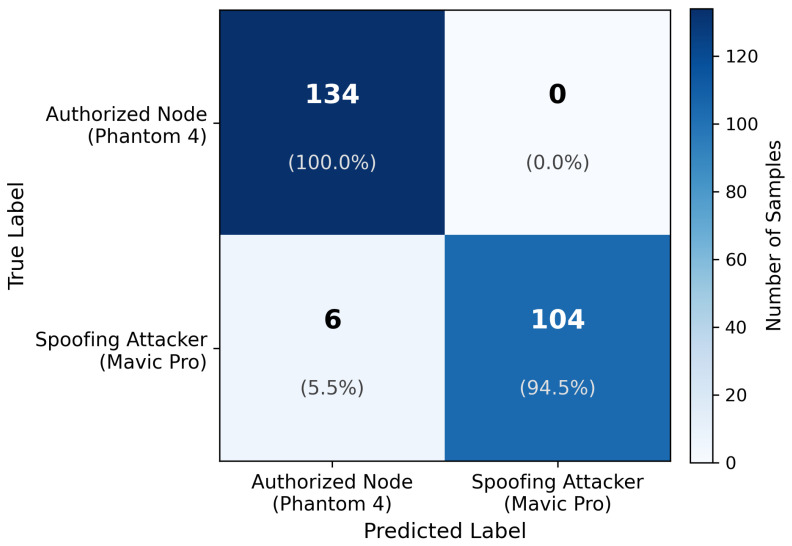
Confusion matrix of the acoustic verification module.

As shown in Figure 4, the acoustic verification module exhibits strong discriminability in distinguishing legitimate nodes from spoofing entities. In a test set consisting of 110 spoofing-attack samples, the model successfully intercepts 104 samples, corresponding to an interception rate of 94.5% and a false acceptance rate of 5.5%. This result is consistent with the experimental conclusion of Diao et al. on UAV authentication using acoustic fingerprints [15]. It also agrees with the effectiveness of acoustic authentication verified by DroneAudioID in open-set authentication scenarios [16]. In addition, Miesikowska’s experiment on UAV classification based on acoustic signals in external environments also supports the distinguishability of rotor acoustic fingerprints [30]. This indicates that even if an attacker successfully clones a digital identity, the intrinsic acoustic features caused by motor speed, propeller structure, and fuselage vibration are difficult to reproduce synchronously, making it difficult for the cloned platform to bypass acoustic verification. Combined with the robustness simulation results in Section 5.3.2, this module still has deployment potential under increased noise, shortened sampling windows, and larger distances.

#### 5.3.2. Acoustic Robustness Experiment

**Figure 5:**
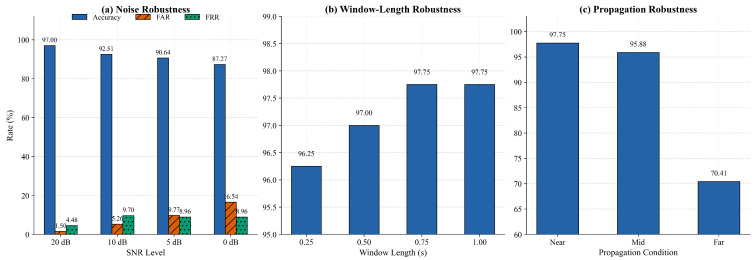
Robustness of the acoustic verification module under noise, short-window, and propagation degradation.

In the file-level robustness experiment based on real audio, this paper evaluates the stability of the acoustic module from three dimensions, namely noise, sampling window, and propagation degradation, using 267 test files from two aircraft models. The corresponding results are shown in Figure 5. First, as shown in Figure 5a, under four noise levels of 20 dB, 10 dB, 5 dB, and 0 dB, the overall accuracy of acoustic verification is 97.00%, 92.51%, 90.64%, and 87.27%, respectively. The corresponding false acceptance rates are 1.50%, 5.26%, 9.77%, and 16.54%, and the false rejection rates are 4.48%, 9.70%, 8.96%, and 8.96%, respectively. The acoustic fingerprint authentication experiment by Diao et al. also shows that a lower signal-to-noise ratio affects UAV acoustic authentication performance [15]. Liu et al.’s study on acoustic recognition in complex low-SNR environments likewise shows that noise is a key factor affecting model robustness [17]. Fan et al.’s research on long-distance UAV sound recognition further indicates that increased propagation distance causes acoustic signal attenuation and increases recognition difficulty [31].

Second, as shown in Figure 5b, considering that the original audio segments are about 1.024 s long, this paper further uses four center-cropping windows of 0.25 s, 0.50 s, 0.75 s, and 1.00 s to evaluate short-duration verification capability. The corresponding accuracies are 96.25%, 97.00%, 97.75%, and 97.75%, respectively. The results show that, for the current data, short windows of 0.50 s or longer can already maintain high recognition accuracy, whereas the 0.25 s window begins to show noticeable degradation. Finally, as shown in Figure 5c, this paper superimposes three levels of propagation degradation, near, medium, and far, on real waveforms to simulate band loss and background interference in open environments. The corresponding accuracies are 97.75%, 95.88%, and 70.41%. These three levels reflect recognition-performance changes caused by deteriorating propagation conditions and do not directly correspond to fixed physical distances. Overall, acoustic verification adapts well to moderate noise and short-window scenarios, but its performance drops significantly under severe propagation degradation. The main bottleneck remains complex environments where low SNR and long-distance propagation jointly affect the signal.

The robustness results show that different artificial disturbances affect verification performance according to how strongly they weaken or mask the rotor harmonic features used by the classifier. General additive noise mainly reduces the SNR and produces gradual degradation rather than abrupt failure. When the added noise level increases from 20 dB to 0 dB, the verifier still retains discriminative capability, although the false acceptance rate increases and the overall accuracy decreases. The strongest degradation appears under the simulated propagation condition, where acoustic components are removed or attenuated over a longer channel. This indicates that major performance loss is more likely when environmental interference, low SNR, and propagation attenuation jointly obscure the rotor harmonic structure used by the classifier. Under these conditions, the acoustic module may generate more false acceptances, and the dynamic trust module must rely more strongly on subsequent authorization evidence to maintain conservative control. The result characterizes robustness under controlled file-level perturbations and identifies the main environmental factors that should be prioritized in future field validation.

### 5.4. Effectiveness Analysis of Bayesian Dynamic Authorization

To verify the decision-making capability of the zero-trust authentication and authorization loop in a dynamic attack-defense environment, we constructed three scenarios under a unified trust threshold and event-triggering rule: additional verification of legitimate nodes, identity-cloning nodes requesting mission-critical privileges, and intermittent spoofing nodes repeatedly probing the system. A discrete-time simulation records the evolution of node trust values over time steps to characterize the practical effects of the system in authorization retention, rapid isolation, and historical penalty memory.

#### 5.4.1. Authorization Retention for Legitimate Nodes

In the legitimate-node authorization-retention scenario, a node triggers one high-risk authorization verification event at time t=20 because of increased mission sensitivity. Two schemes are compared. The first is the proposed scheme, in which SM9 access, acoustic verification, and Bayesian dynamic trust jointly adjust authorization. The second is an SM9 + static trust baseline, which retains only digital access and trust updating under fixed rules, without introducing acoustic verification or Bayesian historical compensation. To avoid separating the authorization simulation from purely rule-based curves, this paper further uses the real acoustic posterior probabilities corresponding to 134 legitimate test files to drive positive evidence updates during the verification phase. This evaluates whether the system can maintain service continuity under additional verification and avoid persistent authorization downgrading for normal nodes.

**Figure 6:**
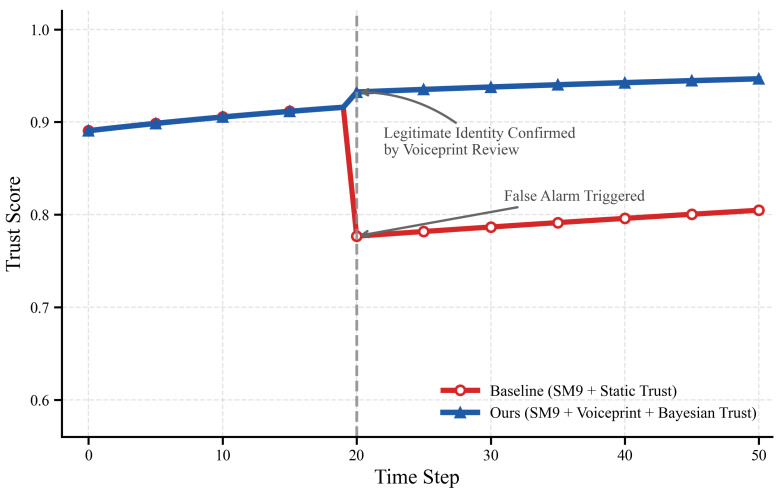
Authorization retention of legitimate nodes.

As shown in Figure 6, before the additional verification is triggered, the average trust values of both schemes rise stably to about 0.916. After the additional verification is triggered at t=20, the average trust value of the SM9 + static trust baseline immediately drops to 0.777 because it lacks physical consistency evidence, and it recovers only slowly to 0.805 over the subsequent 30 time steps, never returning to the pre-verification level. In contrast, the proposed scheme immediately invokes the real acoustic classifier for verification after the verification event is triggered. The average acoustic pass confidence of legitimate nodes is 0.953, and the average trust value of the system remains at 0.932 at the trigger time. It stays above 0.891 throughout the observation window and recovers to 0.947 at the end. This result shows that acoustic verification and Bayesian compensation updating can effectively suppress false privilege reduction and ensure service continuity for legitimate nodes.

#### 5.4.2. Rapid Isolation of Identity-Cloning Nodes

**Figure 7:**
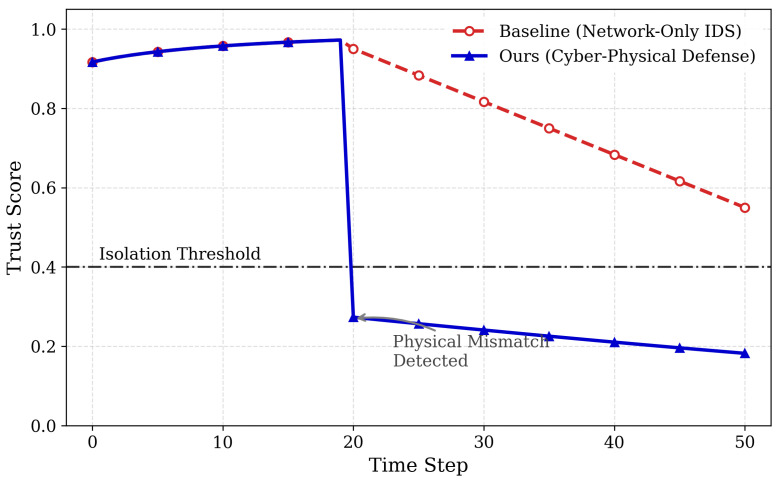
Rapid isolation of identity-cloned nodes.

In the rapid-isolation scenario for identity-cloning nodes, an attacker accesses the network at time t=20 with a stolen legitimate digital identity and immediately requests high-level control privileges. This scenario is used to evaluate the system’s immediate recognition and privilege-reduction capability against attacks in which the digital identity is legitimate, but the physical entity is forged. Figure 7 shows the trust evolution results of two schemes.

As shown in Figure 7, the baseline scheme can complete access authentication, but because it authorizes only according to digital identity or linear reputation, the attacking node’s trust value remains above 0.77 and always higher than the isolation threshold of 0.4. The attacker can therefore remain connected for a relatively long time window. In the proposed scheme, acoustic verification at t=20 immediately detects the entity mismatch. The attacking node’s trust value drops sharply from 0.97 to 0.27 and remains below 0.32 thereafter. This result indicates that acoustic verification can provide a timely veto signal, while Bayesian dynamic authorization further amplifies the suppressive effect of this signal in subsequent decisions.

The same authorization logic is relevant when a legitimate UAV and an impersonating node submit permission requests nearly simultaneously through different gateways. In this case, the gateway does not treat possession of a valid digital credential as sufficient for mission-critical authorization. Duplicated identity use or incompatible access context first marks the session for entity-consistency checking. If the suspect session also produces an acoustic mismatch, this mismatch is used as negative evidence in the dynamic trust state. The session with inconsistent identity and entity evidence is then downgraded or isolated once its trust value falls below the threshold, whereas the session with consistent entity evidence remains subject to normal trust evaluation.

#### 5.4.3. Suppression of Intermittent Spoofing Nodes

**Figure 8:**
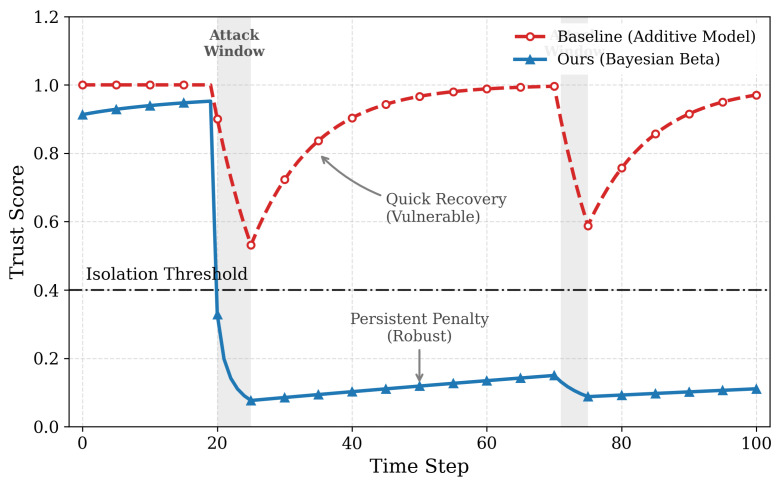
Trust evolution comparison between Bayesian and weighted models.

The intermittent spoofing-node suppression scenario considers a more strategic on-off attack. After being detected, the attacker enters a period of normal behavior and then requests high-risk authorization again, attempting to recover trust through short-term compliance. Figure 8 compares the proposed Bayesian trust model with a traditional weighted model in this scenario.

The simulation results show that during the dormant period from t=25 to t=70, the attacking node stops making high-risk authorization requests and deliberately behaves normally. The trust value of the traditional weighted model quickly rises from 0.39 to 0.81, indicating that the attacker can mask prior malicious behavior through short-term normal behavior. In contrast, the Bayesian model in this paper recovers only to about 0.49 and never re-enters the high-trust region. When the attacker requests high-risk authorizations again after t=75, the Bayesian model retains historical failed-authentication records, causing the trust value to rapidly fall to 0.24 and cross the isolation threshold again. Chae et al.’s study of on-off attacks points out that attackers exploit trust-recovery mechanisms by alternating between normal and malicious behavior to maintain system trust [20]. Mendoza and Kleinschmidt’s IoT experiments also show that retaining direct interaction history helps identify intermittent malicious nodes [21]. Therefore, the results of this paper indicate that the Beta-distribution trust model can more stably support continuous evaluation in zero-trust authorization and reduce the opportunity for intermittent spoofing nodes to restore high privileges through short-term normal behavior.

### 5.5. Ablation Experiment and System Overhead Analysis

#### 5.5.1. Analysis of Ablation Results

To evaluate the independent contribution of each module in the complete framework, this paper further conducts ablation experiments under unified experimental settings. The comparison schemes include four groups: SM9-only, SM9 + acoustic verification, SM9 + Bayesian trust, and the complete scheme. SM9-only retains only the digital identity access mechanism and does not include runtime verification or dynamic trust updating; it is therefore used only to characterize the capability boundary of pure digital access. SM9 + acoustic verification introduces entity verification after access authentication. SM9 + Bayesian trust retains digital access and dynamic authorization but does not use acoustic verification. The complete scheme includes all three mechanisms: digital access, entity verification, and dynamic authorization.

In terms of isolating identity-cloning nodes, the four schemes show clear hierarchical differences. Although SM9-only can block external access requests without a legitimate private key, it cannot distinguish cloned nodes with a legitimate digital identity but inconsistent physical entity when the attacker has stolen a legitimate private key. Therefore, under this attack model, the baseline has an isolation success rate of 0%, and the average isolation time is not applicable. After rotor acoustic verification is added, SM9 + acoustic verification significantly strengthens interception of identity-cloning attacks. The isolation success rate rises to 93.4%, and blocking is triggered after an average of 2.3 time steps. SM9 + Bayesian trust has historical penalty capability, but because it lacks entity evidence, it still suffers from delayed early recognition of digital identity cloning. Its isolation success rate is 77.1%, and it requires an average of 4.8 time steps to fall below the isolation threshold. In contrast, the complete scheme introduces acoustic verification for high-risk requests and uses Bayesian memory to complete subsequent authorization adjustment. It further increases the isolation success rate to 96.5% and crosses the isolation threshold after an average of 1.6 time steps.

For authorization retention of legitimate nodes and suppression of intermittent attacks, Bayesian dynamic trust plays a more prominent role. For SM9-only, because it does not participate in runtime privilege reduction or recovery, the unnecessary authorization reduction rate and authorization recovery time of legitimate nodes are not applicable. This also means that it cannot adjust privileges in a fine-grained manner according to risk changes during the session phase. By comparison, the unnecessary authorization reduction rates of SM9 + acoustic verification and SM9 + Bayesian trust are 7.6% and 6.3%, respectively, while their authorization recovery times are 3.2 and 2.6 time steps. The complete scheme controls the unnecessary authorization reduction rate at 3.4% and recovers stable authorization within 1.4 time steps. For on-off intermittent spoofing attacks, if Bayesian dynamic trust is absent, the system can still rely on acoustic recognition to identify physically inconsistent nodes at critical moments, but it cannot form persistent memory of historical malicious behavior. After Bayesian dynamic trust is introduced, the trust-recovery slope of attacking nodes becomes significantly slower. In the complete scheme, when the attacker requests high-risk authorizations again, the system can more rapidly suppress its long-term authorization level.

Table 8 summarizes the results of the four ablation schemes in identity-cloning isolation, legitimate-node authorization retention, and intermittent-attack suppression. Here, RDE denotes risk-mitigation effectiveness, and RIS denotes the average number of time steps required for a risk node to fall below the isolation threshold.

Overall, SM9 establishes a low-overhead digital identity anchor, acoustic verification provides entity consistency evidence, and Bayesian dynamic trust converts single authentication conclusions into a continuous authorization-updating process. The ablation results show that these three mechanisms are not simply additive. Without acoustic evidence, the system cannot promptly identify cloned nodes that hold legitimate credentials. Without Bayesian dynamic trust, the system cannot stably suppress intermittent spoofing or unnecessary authorization-reduction fluctuations. Only when the three mechanisms cooperate can the system simultaneously achieve rapid isolation, authorization recovery, and long-term suppression.

#### 5.5.2. System Overhead and Efficiency Analysis

For system efficiency, this paper compares the resource cost of the complete scheme with that of continuous acoustic authentication. The complete scheme keeps SM9 as the normal access mechanism and invokes acoustic verification only under high-risk authorization triggers or abnormal states. Its extra overhead therefore mainly comes from on-demand recording, feature extraction, and classification inference. This design avoids always-on high-load authentication.

The evaluation metrics include authentication activation frequency, average single-step inference latency, normalized energy consumption, and GPU memory usage. Compared with continuous acoustic authentication, the complete scheme reduces authentication activation frequency from 100.0% to 13.2%, average single-step inference latency from 52.4 ms to 8.6 ms, normalized energy consumption from 1.00 to 0.17, and GPU memory usage from 1.85 GB to 0.42 GB. The reductions are 86.8%, 83.6%, 83.0%, and 77.3%, respectively. The overhead and resource-consumption comparison is summarized in Table 9. These results suggest that the on-demand verification mechanism can significantly reduce operating cost without sacrificing key security benefits.

From a deployment perspective, SM9-only has the lowest access complexity, but it cannot provide continuous identity assurance during the session phase. SM9 + acoustic verification improves security, but continuous acoustic checking would increase recording, feature extraction, and inference overhead. SM9 + Bayesian trust has low runtime cost, but it lacks entity evidence and remains weak against identity cloning. The complete scheme invokes acoustic verification only on triggered events and uses Bayesian dynamic trust to reduce unnecessary high-frequency authentication. It therefore achieves higher security benefit per unit resource cost.

### 5.6. Comparative Analysis

Existing UAV authentication and trust-management studies differ in research objects, threat assumptions, and metrics. Access-authentication studies address access legitimacy. Entity identification studies examine whether the current flight platform matches the registered entity. Dynamic-trust studies characterize runtime risk states. Following this security chain, this paper selects four representative works that match the main modules of the proposed scheme: Xie and Wang for digital identity access, DroneAudioID for rotor acoustic verification, Zhang et al. for RF-spectrum-based authentication with zero-trust principles, and GDM-DTM for runtime dynamic trust judgment. Table 10 compares functional coverage. Table 11 then compares authentication latency, communication overhead, entity-verification performance, risk-mitigation effectiveness, risk-isolation speed, and on-demand triggering efficiency. PUF-based schemes strengthen device-side key material or challenge-response non-clonability. However, their evidence is mainly used for access authentication and does not re-check acoustic, visual, or behavioral features of the in-flight platform. Therefore, entity verification is marked as partial support.

In Table 10, DIA denotes digital identity authentication, PEA denotes entity verification, and DA denotes dynamic authorization. “✓”, “△”, and “×” denote full support, partial support, and no support, respectively. The selected schemes each emphasize one security link. They do not simultaneously cover identity confirmation before access, entity verification after access, and authorization adjustment during the session. Xie and Wang focus on access legitimacy, DroneAudioID focuses on acoustic entity differentiation, Zhang et al. combine RF-spectrum-based entity authentication with zero-trust gatekeeping, and GDM-DTM focuses on runtime malicious-node detection. By contrast, the proposed scheme integrates digital identity, entity evidence, and dynamic authorization state into one updating process. It can continuously constrain nodes that hold valid credentials but have inconsistent physical entities, as well as nodes whose behavior becomes abnormal after authentication.

In Table 11, $T_{\mathrm{auth}}$ denotes representative authentication or cross-domain authentication latency, $C_{\mathrm{comm}}$ denotes communication overhead converted uniformly into bytes, and SPEA denotes the representative entity verification accuracy or interception rate. RDE denotes risk-mitigation effectiveness; for GDM-DTM, it refers to the reported malicious-node detection accuracy, whereas for the proposed scheme, it refers to the identity-cloning node isolation success rate. RIS denotes the average number of time steps required for a risk node to fall below the isolation threshold. AF denotes the activation frequency of acoustic verification, and $E_{\mathrm{red}}$ denotes the normalized energy reduction relative to continuous acoustic authentication. Since the selected studies evaluate different security links, these indicators should be read as link-specific evidence of efficiency, recognition capability, and authorization adjustment rather than as a single homogeneous benchmark.

For digital identity access, Xie and Wang report a cross-domain authentication latency of 20.01 ms and a communication overhead of about 160 bytes. This reflects the efficiency of PUF-based authentication in lightweight access scenarios. The proposed SM9 three-message access authentication has an average latency of 20.4 ms and a communication overhead of 152 bytes. Its latency is comparable, and its communication payload is slightly lower. Thus, the proposed access module keeps low computation and communication overhead while introducing a traceable identity anchor and bidirectional identity confirmation. It also provides a linkable digital identity basis for later acoustic verification and dynamic trust updating.

For entity verification and runtime authorization, the main advantage of the proposed scheme is the continuous use of multi-source evidence. DroneAudioID achieves 99.6% accuracy in an independent acoustic authentication task. This is higher than the 94.5% spoofing-entity interception rate of the proposed on-demand short-window verification. This gap shows that a scheme optimized only for acoustic classification can achieve higher single-task accuracy. Zhang et al. report 85.67% closed-set accuracy for RF-spectrum-based UAV type classification using a 0.23M-parameter lightweight network. The proposed scheme achieves a higher SPEA of 94.5%, but it should be noted that the two tasks differ: Zhang et al. classify UAV types from RF signals, whereas the proposed scheme detects spoofing entities from rotor acoustic features. However, the proposed scheme does not stop at one-time classification. It uses the acoustic result as physical evidence for dynamic trust updating. In the identity-cloning experiment, the complete scheme achieves an RDE of 96.5%, higher than the 85.04% malicious-node detection accuracy reported by GDM-DTM, and its RIS is 1.6 time steps. The on-demand triggering mechanism also reduces the activation frequency of acoustic verification to 13.2% and reduces normalized energy consumption by 83.0% compared with continuous acoustic authentication. Therefore, the proposed scheme is not superior on every isolated metric. Its advantage lies in coordinated updating among access identity, entity evidence, and authorization state. It keeps access overhead low, improves risk convergence, strengthens authorization control, and reduces the cost of continuous acoustic authentication.

The experimental results allow us to identify each module’s contribution to the overall framework. SM9 access authentication establishes the digital identity anchor with low overhead (20.4 ms, 152 bytes) but cannot detect credential theft or platform cloning. Acoustic verification addresses this gap, achieving a 94.5% spoofing-entity interception rate, though its standalone accuracy (94.5%) is lower than that of a dedicated acoustic classifier (99.6%), reflecting the trade-off imposed by on-demand short-window sampling for energy efficiency. The Bayesian dynamic trust model converts acoustic evidence into authorization decisions, achieving a 96.5% isolation success rate with 1.6-step convergence—higher than the 85.04% reported by a standalone trust model—because it integrates authentication, verification, and behavioral evidence rather than relying on a single source. The on-demand triggering mechanism reduces acoustic activation frequency to 13.2% and energy consumption by 83.0%, demonstrating that risk-driven selective activation preserves security effectiveness while significantly reducing resource overhead. These results indicate that the primary driver of performance gains is the coordinated updating among the three security layers, rather than any individual module in isolation.

The results also show the engineering boundaries of the proposed scheme. First, the access latency and communication overhead are close to those of a representative lightweight authentication scheme, but the results are mainly from protocol-level tests. Onboard cryptographic implementation, wireless transmission, and gateway scheduling may still affect end-to-end overhead. Second, the 94.5% interception rate of the proposed acoustic verification is lower than the 99.6% accuracy of the specialized acoustic authentication scheme. This indicates that on-demand short-window acoustic verification still needs better robustness in complex environments. Strong wind, rain, urban background noise, simultaneous multi-UAV flight, reflection, reverberation, flight-attitude changes, and motor or propeller wear may change the acoustic feature distribution. These factors can affect the discrimination of same-model spoofing platforms. Field data, onboard deployment, and long-term testing are therefore needed to further verify the operational limits of the proposed closed-loop mechanism.

## 6. Conclusions

This paper presents a zero-trust authentication and authorization framework for UAV systems by integrating SM9 access, rotor acoustic fingerprint verification, and Bayesian dynamic trust evaluation. SM9 provides a lightweight and traceable digital identity anchor, rotor acoustic fingerprints provide runtime entity evidence, and Bayesian trust updating converts access, verification, and behavioral evidence into authorization adjustments. Access authentication, entity verification, and dynamic authorization thus form a continuous decision loop rather than separate security checks.

The experimental results show that the average latency of the SM9 three-message access authentication is 20.4 ms, with a communication overhead of 152 bytes. The acoustic verification module achieves a 94.5% interception rate on spoofing samples. For identity-cloning nodes, the complete scheme reaches a 96.5% isolation success rate and pushes attacking nodes below the isolation threshold after an average of 1.6 time steps, while keeping the unnecessary authorization reduction rate of legitimate nodes at 3.4%. Compared with continuous acoustic authentication, the on-demand triggering mechanism reduces acoustic verification activation frequency to 13.2% and lowers normalized energy consumption by 83.0%. These results suggest that the framework can constrain credential-bearing but physically inconsistent nodes with low access overhead and suppress fast trust recovery under intermittent spoofing behavior.

The study also suggests several directions for further work. Future research should validate the closed-loop mechanism on real UAV platforms and during long-term field operation. It should also reduce protocol and implementation overhead on resource-constrained devices and improve acoustic verification in low-SNR, multi-UAV, weather-affected, and reverberant environments. Combining rotor acoustic fingerprints with additional sensing evidence may further improve robustness without returning to continuous high-cost authentication. Future research should also explore the applicability of the proposed framework to wearable-UAV and body-UAV communication scenarios, where power budgets, body-area interference, and proximity-based trust assumptions introduce additional constraints beyond those considered in this work.

## Figures and Tables

**Table 1:** Overall notations for the system model.

Symbol	Definition and Description
*U* = $u_{1}$, $u_{2}$, …, $u_{N}$	Set of UAV nodes in the UAV mission system; N denotes the number of nodes.
$u_{i}$, $u_{j}$	Any two UAV nodes in the UAV mission system.
*T* = 1, 2, …, $T_{\max}$	Discrete monitoring horizon; Tmax denotes the maximum time slot.
*t*	Current discrete time slot.
$A_{i}\left(t\right)$	Digital-identity authentication evidence of node $u_{i}$ at time *t*.
$R_{i}\left(t\right)$	Entity verification evidence of node $u_{i}$ at time *t*.
$B_{i}\left(t\right)$	Authorization-behavior evidence of node $u_{i}$ at time *t*.
$I_{i}^{trig}\left(t\right)$	Acoustic fingerprint verification trigger variable; 1 indicates triggering and 0 otherwise.

**Table 2:** Notations for the SM9-based mutual authentication protocol.

Symbol	Definition and Description
KGC	Key generation center responsible for system master-key generation and private-key provisioning.
$G_{1},G_{2},G_{T},e,P_{2}$	Bilinear groups, pairing, and generator used in SM9 system initialization.
$N,s,P_{pub}$	Group order, system master private key, and master public key.
u,g	UAV node and gateway participating in mutual authentication.
$ID_{u},ID_{g}$	Identity identifiers of the UAV and gateway.
$d_{s,u},d_{s,g}$	Private keys generated by the KGC for the UAV and gateway identities.
SE	On-board secure element used to store the UAV private key.
$R_{u},R_{g}$	Random challenges generated by the UAV and gateway.
$M_{req},M_{resp},M_{conf}$	Authentication request, gateway response, and UAV confirmation messages.
$\sigma_{g},\sigma_{u}$	Gateway response signature and UAV confirmation signature.
Sign(·),Verify(·)	SM9 signature generation and verification algorithms.
$A_{i}\left(t\right)$	SM9 access result used as digital-identity evidence.

**Table 3:** Notations for acoustic-fingerprint-based entity verification.

Symbol	Definition and Description
$ID_{U}$	Claimed digital identity of the UAV.
$V_{U}$	Registered acoustic fingerprint template bound to UAV identity $ID_{U}$.
$S_{i}^{raw}\left(t\right)$	Short-window raw rotor-acoustic signal captured at time *t* by the monitoring terminal.
$x_{i}^{phy}\left(t\right)$	Current acoustic feature vector extracted using MFCC.
MFCC	Mel-frequency cepstral coefficients describing the rotor-sound spectral envelope.
$I_{i}^{trig}\left(t\right)$	Acoustic fingerprint verification trigger variable.
$R_{i}\left(t\right)$	Entity verification result; 1 denotes a match and 0 denotes a mismatch.

**Table 4:** Notations for Bayesian trust evaluation and dynamic authorization.

Symbol	Definition and Description
$\alpha_{i}\left(t\right),\beta_{i}\left(t\right)$	Accumulated benign and malicious evidence of node $u_{i}$ at time *t*.
$\Delta \alpha_{t},\Delta \beta_{t}$	Positive and negative evidence increments converted in the current time slot.
λ	Temporal decay factor used to discount historical evidence.
$\omega_{res},\omega_{pun}$	Trust-recovery reward weight and malicious-behavior penalty weight.
${Trust}_{i}\left(t\right)$	Bayesian trust value of node $u_{i}$ at time *t*.
$\theta_{th}$	Trust-decision threshold; nodes below this value are isolated.
$D_{i}\left(t\right)$	Dynamic authorization decision of node $u_{i}$ at time *t*.
$C_{i}\left(t\right)$	Authentication, verification, and authorization cost of node $u_{i}$ at time *t*.
$B_{total}$	Total computation or energy budget allowed by the system.
$y_{i}\left(t\right)$	Expected authorization label or ground-truth state of node $u_{i}$ at time *t*.

**Table 5:** Experimental settings and authentication-authorization scenario parameters.

Parameter	Setting/Description
GPU	NVIDIA GeForce RTX 3090 (24 GB memory)
CPU	Intel Core i9 processor
Software environment	Python 3.8
Audio processing library	Librosa
Acoustic feature	MFCC rotor-acoustic feature
Initial trust parameters	α=1, β=1; initial trust value = 0.5
Trust isolation threshold	0.4

**Table 6:** Simulation overhead of the SM9 access module.

Metric	Mean (ms)	P95 (ms)	Description
UAV-side SM9 signing	8.42	10.15	Protocol-level simulation for generating the Mconf confirmation signature.
Gateway-side SM9 verification	11.76	14.28	Protocol-level simulation for verifying the UAV signature.
Gateway-side SM9 signing	7.95	9.63	Protocol-level simulation for generating the Mresp challenge-response signature.
UAV-side SM9 verification	12.31	14.96	Protocol-level simulation for verifying the gateway signature.
Complete three-message handshake	20.40	24.30	Protocol-level simulation excluding wireless queuing and transmission delay; P95 estimated using the original mean-to-P95 ratio.

**Table 7:** Simulated communication overhead of SM9 access.

Message	Fields	Estimated Size (Bytes)
Mreq	UAV ID + Ru	32
Mresp	Gateway ID + Rg + SM9 signature	60
Mconf	UAV ID + Rg + SM9 signature	60
Total	Mreq + Mresp + Mconf	152

**Table 8:** Ablation results of the proposed authentication and authorization scheme.

Scheme	RDE (%)	RIS (Step)	Unnecessary Authorization Reduction Rate (%)	Authorization Recovery Time (Steps)	On-Off Attack Suppression
SM9-only	0	--	--	--	Not supported
SM9 + acoustic verification	93.4	2.3	7.6	3.2	Weak; no historical memory
SM9 + Bayesian trust	77.1	4.8	6.3	2.6	Moderate; with historical penalty
Full scheme	96.5	1.6	3.4	1.4	Strong; combines entity evidence and historical memory

**Table 9:** Comparison of system computational overhead and resource consumption.

Metric	Continuous Acoustic Authentication	Dynamic Authorization-Triggered Authentication	Reduction
Activation frequency (%)	100.0	13.2	86.8%
Average latency (ms)	52.40	8.60	83.6%
Normalized energy (relative unit)	1.00	0.17	83.0%
GPU memory (GB)	1.85	0.42	77.3%

**Table 10:** Functional coverage of representative UAV security schemes.

Scheme	DIA	PEA	DA
Xie and Wang (2025) [9]	✓	△	×
DroneAudioID (2025) [16]	×	✓	×
Zhang et al. (2025) [27]	✓	✓	×
GDM-DTM (2025) [22]	×	×	✓
Proposed scheme	✓	✓	✓

**Table 11:** Key performance indicators of selected representative UAV security schemes.

Scheme	$T_{\mathrm{auth}}$ (ms)	$C_{\mathrm{comm}}$ (Byte)	SPEA (%)	RDE (%)	RIS (Step)	AF (%)	$E_{\mathrm{red}}$ (%)
Xie and Wang (2025) [9]	20.01	160	--	--	--	--	--
DroneAudioID (2025) [16]	--	--	99.6	--	--	--	--
Zhang et al. (2025) [27]	--	--	85.67	--	--	--	--
GDM-DTM (2025) [22]	--	--	--	85.04	--	--	--
Proposed scheme	20.4	152	94.5	96.5	1.6	13.2	83.0

## Data Availability

The data and code supporting the findings of this study are available from the corresponding author upon reasonable request.
